# Virtual Visits and Patient-Centered Care: Results of a Patient Survey and Observational Study

**DOI:** 10.2196/jmir.7374

**Published:** 2017-05-26

**Authors:** Kimberlyn Marie McGrail, Megan Alyssa Ahuja, Chad Andrew Leaver

**Affiliations:** ^1^ Centre for Health Services and Policy Research Vancouver, BC Canada; ^2^ Canada Health Infoway Toronto, ON Canada

**Keywords:** virtual visits, telehealth, primary care delivery, patient-centered care

## Abstract

**Background:**

Virtual visits are clinical interactions in health care that do not involve the patient and provider being in the same room at the same time. The use of virtual visits is growing rapidly in health care. Some health systems are integrating virtual visits into primary care as a complement to existing modes of care, in part reflecting a growing focus on patient-centered care. There is, however, limited empirical evidence about how patients view this new form of care and how it affects overall health system use.

**Objective:**

Descriptive objectives were to assess users and providers of virtual visits, including the reasons patients give for use. The analytic objective was to assess empirically the influence of virtual visits on overall primary care use and costs, including whether virtual care is with a known or a new primary care physician.

**Methods:**

The study took place in British Columbia, Canada, where virtual visits have been publicly funded since October 2012. A survey of patients who used virtual visits and an observational study of users and nonusers of virtual visits were conducted. Comparison groups included two groups: (1) all other BC residents, and (2) a group matched (3:1) to the cohort. The first virtual visit was used as the intervention and the main outcome measures were total primary care visits and costs.

**Results:**

During 2013-2014, there were 7286 virtual visit encounters, involving 5441 patients and 144 physicians. Younger patients and physicians were more likely to use and provide virtual visits (*P*<.001), with no differences by sex. Older and sicker patients were more likely to see a known provider, whereas the lowest socioeconomic groups were the least likely (*P*<.001). The survey of 399 virtual visit patients indicated that virtual visits were liked by patients, with 372 (93.2%) of respondents saying their virtual visit was of high quality and 364 (91.2%) reporting their virtual visit was “very” or “somewhat” helpful to resolve their health issue. Segmented regression analysis and the corresponding regression parameter estimates suggested virtual visits appear to have the potential to decrease primary care costs by approximately Can $4 per quarter (Can –$3.79, *P*=.12), but that benefit is most associated with seeing a known provider (Can –$8.68, *P*<.001).

**Conclusions:**

Virtual visits may be one means of making the health system more patient-centered, but careful attention needs to be paid to how these services are integrated into existing health care delivery systems.

## Introduction

A greater orientation to information technology in primary care opens new possibilities for health care delivery, one of which is virtual care. “Virtual care” is a broad term meant to capture all clinical interactions in health care that do not involve the patient and provider being in the same room at the same time [1]. Consultations may be asynchronous, whereby patients answer structured clinical questions online and then receive care from a physician at a later time (“e-visits”), or synchronous, whereby patients interact with physicians in real time via telephone (“teleconsultations”), videoconference (“virtual visits”) [2-5], or even by text [6].

Canada is a geographically large country, and physicians are disproportionately clustered within urban and semiurban settings, with known shortages of primary care providers in more rural and remote areas [7]. However, limited access to primary care is not a feature of rural areas only. There are well-documented accessibility issues in urban areas as well, whereby many patients do not have a regular primary care physician or cannot access their physician for in-person appointments within a timeframe that meets their current needs [8-10]. New modes of contact, including virtual visits, are one potential way to solve at least some of these access issues.

Some health systems are integrating virtual care into primary care practices as a complement to existing modes of care. The US health care provider Kaiser Permanente has altered their delivery of health care services to improve information continuity and to provide easy access to appropriate care for patients through initiatives such as electronic messaging with the care team, scheduled telephone visits, and a comprehensive patient portal [11]. This is, at least in part, a response to a growing focus on patient-centered care that aims to provide care that is both “accessible” and “acceptable” [12,13]. Virtual care can, for example, increase access to care for individuals who have difficulty presenting in-person for primary care services, such as those living in long-term care facilities and/or those with mobility issues [14].

Virtual care is also understood as a means of making care more convenient to patients and is gaining momentum in the United States and other advanced health systems alongside other innovations such as retail clinics and other forms of walk-in care [15-20]. There is, at present, limited empirical evidence about how patients view virtual care and more specifically virtual visits, how such care affects overall primary care use, and whether integration of virtual visits in existing relationships or more as a “walk-in” service matters. This research aims to fill that gap, focusing on British Columbia, Canada, a province with a population of 4.5 million people, which has had public funding of virtual visits since 2012.

This study has both descriptive and analytic objectives. The descriptive objectives are to assess the users and providers of virtual visits, as well as the reasons patients give for their use. The analytic objective is to assess empirically the influence of the use of virtual visits on overall primary care use and costs, paying specific attention to whether virtual care is provided by a known or a new primary care physician.

## Methods

This was a mixed-methods study that included a patient survey and an analysis of administrative health care data. The study was undertaken in British Columbia, Canada, a province with universal health care coverage for a population of approximately 4.5 million. IRB-Services Canada provided research ethics approval for the patient survey, and the University of British Colombia Behavioural Research Ethics Board for the observational study.

### Observational Study

#### Data Sources

We used data for fiscal years 2010/2011 to 2013/2014. The following files were linked:

Medical Services Plan (MSP) Payment Information File: this file includes all fee-for-service payment data for physicians and contains the information needed to identify virtual visit encounters between physicians and patients [21];

Consolidation file (MSP Registration and Premium Billing): this file includes patient characteristics for all BC residents who are eligible and receive publicly funded health care services, including patient demographic, location, and socioeconomic status (SES) information based on neighborhood income [22];

PharmaNet: this file includes all prescriptions filled in British Columbia, regardless of payment source. This file was used in a measure to classify physician practice style and to examine the frequency of prescribing as part of virtual visits [23]; and

MSP Practitioner Information File: this file includes demographic, specialty, location of training, and location of practice of all primary care providers in British Columbia [24].

We used the Johns Hopkins’ adjusted clinical group (ACG) case-mix system to assess patients’ morbidity burden. This system uses a risk-adjustment methodology to describe and predict expected use and cost of health care services and has been validated for use with BC administrative data [25]. More specifically, we used aggregated diagnosis groups (ADGs), a midlevel grouping based on diagnosis codes in health care encounters, to assess comorbidities. There are 32 ADGs in the overall system, eight of which are considered major; we used a count of major ADGs as our measure of health status [26].

#### Defining the Cohort and Comparison Group

The cohort was everyone who had one or more virtual visit fee codes billed on their behalf at any point in the study period. The population comparison group used for initial descriptive analyses were all other BC residents.

A second (and main) comparison group was matched (3:1) on 5-year age group, sex, health service delivery area (HSDA) of residence (there are 16 HSDAs in British Columbia), and number of major ADGs. This comparison group enabled analysis of the effects of virtual visits on overall patterns of health care services use. We anchored the match so that the comparison group had a primary care encounter at approximately the same time as the case’s virtual visit. This was done because a virtual visit will naturally create a spike in use because it represents an individual’s decision to access the health care system. Matching based on that access creates a similar spike in the comparison group and thus increases the likelihood that any differences preceding or following that spike were attributable to the virtual visit.

#### Other Variables and Analysis

Our outcome of interest was costs associated with primary care visits. We used broader physician data to assess referrals to specialists and for laboratory and imaging services, but these aspects of care were not included in the cost outcome because, in the BC context, primary care physicians do not typically provide in-office laboratory or imaging services. Descriptive analyses assessed the age, sex, location, and morbidity distribution of individuals who had virtual visits, and the specialty, age, and sex of primary care physicians who provided them. We further separated users into those who saw a provider with whom they have had a traditional office visit versus seeing a new provider. Referrals and prescriptions within 30 days of a visit (except 90 days for imaging to allow for longer wait times) were captured using physician and pharmaceutical data.

Primary care physician practice style was classified into three groups—high responsibility, mixed practice, and low responsibility—based on a cluster analysis using five variables derived from fee-for-service payment data. This approach was developed previously [27] and was shown to create distinct groups, with high-responsibility physicians providing more comprehensive or full-service care and low-responsibility physicians providing care more consistent with a walk-in clinic-style practice.

Time series analysis was used to assess the effect of virtual visits on overall visits with primary care physicians. A time series approach helps identify changes in both the trend (slope) in service use and amount (level) of service use before and after an initial virtual visit encounter. No change in amount of service will be interpreted as virtual visits serving a substitute function for other health care services use.

More specifically, we will analyze time series data using segmented regression [28] in the form:

Y_jkt_=β_0_+β_1_×time_t_+β_2_×group_k_+β_3_×group_k_×time_t_+β_4_×level_jt_+ β_5_×trend_jt_+β_6_×level_jt_×group_k_+β_7_×trend_jt_×group_k_+ε_jkt_

where *Y* is the mean number of primary care visits / costs per month, *j* is the intervention status, *k* is the group, and *t* represents time. In these models, *β*_0_ is the intercept and *β*_1_ is the existing trend in the matched comparison group, *β*_2_ estimates the preintervention difference in level between the intervention group and its matched controls, and *β*_3_ estimates the difference in trend, and *β*_4_ and *β*_5_ estimate the changes in level and trend for matched controls postintervention, respectively. The real parameters of interest are β_6_ and β_7_ because they estimate the difference in level (*β*_6_) and trend (*β*_7_) between the intervention group and matched controls following the intervention. Statistically significant values for these latter two parameters would indicate that the use of telemedicine had an effect on primary care services use. Analyses were completed using the autoreg procedure in SAS 9.0 and were assessed for autocorrelation.

### Patient Survey

#### Recruitment and Analysis

Inclusion criteria for the patient survey were 18 years of age or older, had at least one virtual visit with a primary care physician in the past 6 months, currently living in British Columbia, and able to complete the survey in English. A total of 3025 patients were deemed eligible. The virtual visit technology provider issued an electronic invitation to participate in the survey and a single reminder to all eligible registrations. No monetary incentives were offered, but patients who completed the full survey were entered into a draw to win a prize (worth up to Can $500) identified by Harris-Decima, the survey company. Informed consent was obtained electronically before commencing the survey. Analyses focused on descriptive statistics, including univariate and bivariate analyses with tests of statistical significance. Analyses were conducted with SPSS version 21.

## Results

### Observational Study

**Figure 1:**
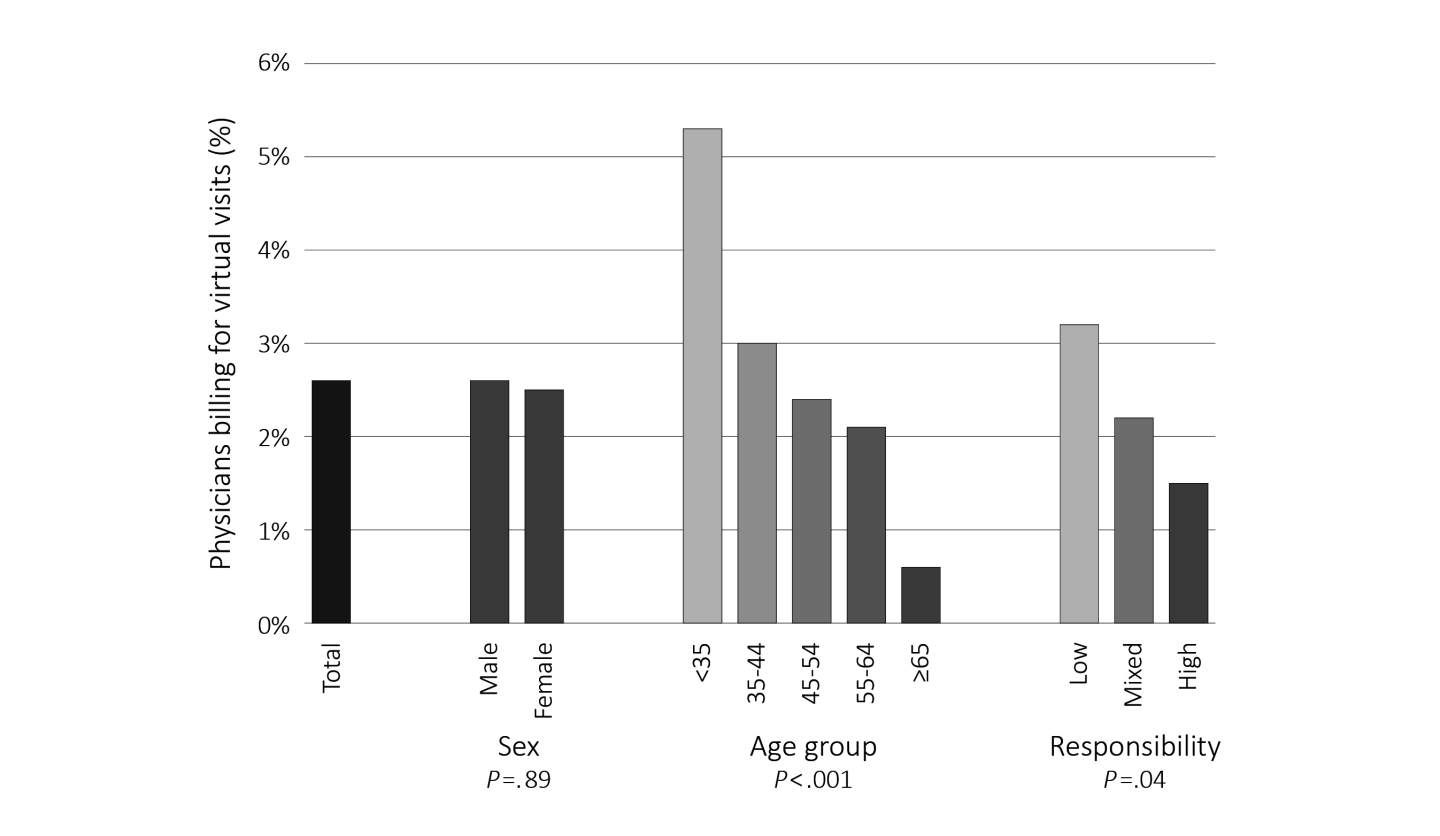
Percentage of primary care physicians who billed for virtual visits by sex, age group, and responsibility level in 2013/2014.

In 2013/14, 144 of 5598 primary care physicians provided at least one virtual visit, or 2.57% of the total primary care physician population (Figure 1). Male and female providers were equally likely to provide these services (*P*=.89). There were physicians in every age group providing virtual visits, although the likelihood decreased monotonically with increasing age—from 5.3% (33/624) of physicians younger than 35 years to 0.8% (6/724) of those aged 65 years and older (*P*<.001). Physicians defined as low responsibility (ie, practices appear to be walk-in style) were the most likely to bill virtual visit fee items (3.24%, 51/1574), whereas those defined as high responsibility were least likely (1.40%, 14/1000, *P*=.04).

There were 5441 patients who had at least one virtual visit, which equates to 105.45 people per 100,000 of the provincial population. Table 1 shows demographics and health status of virtual visit users, both in numbers and as a percentage of the total population. Use of these services was highest in the 20 to 44 years age group (53.45%, 2908/5441) and lowest among those 85 years and older (0.77%, 42/5441). Use was highest (in percentage of population terms) in the Northern Health Authority (11.27%, 613/5441), a largely rural and remote part of the province, but the next highest rates of use were in the two most urbanized regions—Vancouver Coastal (24.74%, 1346/5441) and Fraser Health (45.07%, 2452/5441).

**Figure 2:**
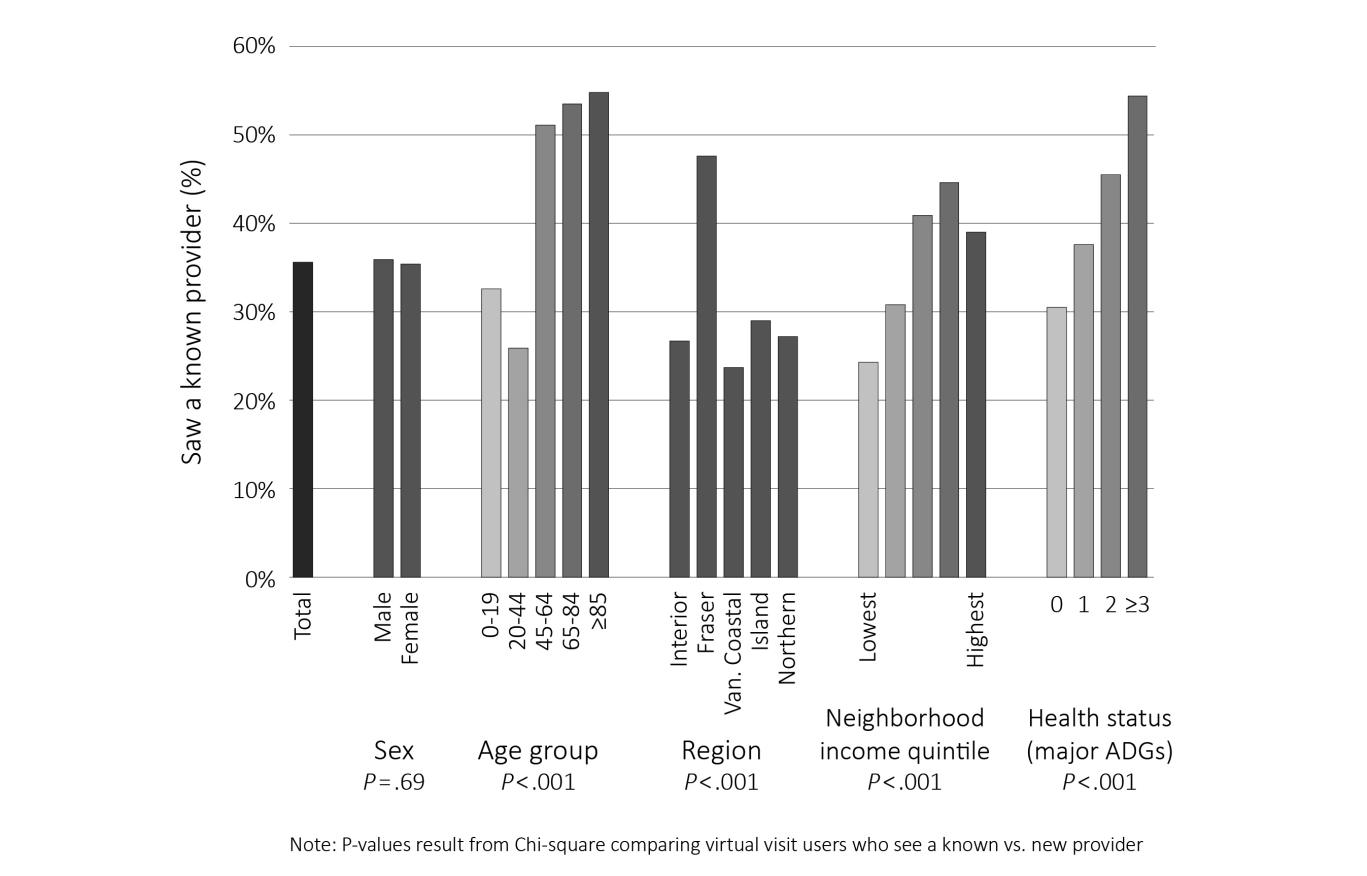
Percentage of patients who saw a known provider for first virtual visit, by sex, age group, region, neighborhood income, and health status in 2013/2014.

**Table 1:** Demographics of virtual visit users (cohort), the BC total population (comparison group), and traditional primary care users (matched control).

Demographics		Cohort, n (%)	Comparison group, n (%)	Matched control, n (%)	Virtual visit users per 100,000 population, n
N		5441	5,154,164^a^	21,176	105.45
**Sex**					
	Male	2246 (41.28)	2,567,271 (49.81)	6484 (41.21)	87.41
	Female	3195 (58.72)	2,585,293 (50.16)	9251 (58.79)	123.43
**Age group (years)**					
	0-19	668 (12.28)	1,041,727 (20.21)	1816 (11.54)	64.08
	20-44	2908 (53.45)	1,759,334 (34.13)	8249 (52.42)	165.02
	45-64	1356 (24.92)	1,454,896 (28.23)	4127 (26.23)	93.12
	65-84	467 (8.58)	735,034 (14.26)	1402 (8.91)	63.49
	≥85	42 (0.77)	163,173 (3.17)	141 (0.90)	25.73
**Region**					
	Interior	558 (10.26)	821,389 (15.94)	1596 (10.16)	67.89
	Fraser	2452 (45.07)	1,804,191 (35.00)	7150 (45.53)	135.72
	Vancouver Coastal	1346 (24.74)	1,287,575 (24.98)	3850 (24.52)	104.43
	Island	459 (8.44)	833,223 (16.17)	1343 (8.55)	55.06
	Northern	613 (11.27)	318,779 (6.18)	1764 (11.23)	191.93
**Income quintile**					
	Lowest	1065 (20.31)	1,028,089 (20.42)	3326 (21.42)	103.48
	2	968 (18.46)	1,022,091 (20.30)	3312 (21.33)	94.62
	3	1038 (19.79)	1,014,178 (20.14)	3183 (20.50)	102.24
	4	1220 (23.26)	1,007,627 (20.01)	3020 (19.45)	120.93
	Highest	953 (18.17)	963,518 (19.13)	2686 (17.30)	98.81
**Health status**					
	0 major ADGs	3044 (55.95)	3,648,817 (70.79)	8699 (55.28)	83.35
	1	1451 (26.67)	956,439 (18.56)	4297 (27.31)	151.48
	2	569 (10.46)	330,074 (6.40)	1645 (10.45)	172.09
	3+	377 (6.93)	218,834 (4.25)	1094 (6.95)	171.98

^a^ The total population that lived in British Columbia at any time during 2010/2011 to 2013/2014.

When limited to the first virtual visit per person, just over one-third (35.58%, 1936/5441) of those visits were with providers already known to the patient and two-thirds (64.42%, 3505/5441) were with a new provider (Figure 2). Males and females were equally likely to have seen their physician before their virtual visit (*P*=.69). People older than 45 years, those who lived in the Fraser Health Authority (largest urban health authority), and those who had more complex health conditions (≥3 major ADGs) were the most likely to have seen their virtual visit provider previously in a nonvirtual setting (*P*<.001). There was no clear pattern by SES, but those in the lowest quintile were the least likely to have seen their virtual visit provider previously (24.32%, 259/1065; *P*<.001).

#### Virtual Visit Users Who Saw a Known Versus a New Provider

Table 2 provides the diagnoses for patients who had a virtual visit in the study period compared to the matched controls. A larger proportion of BC patients with a virtual visit sought care for a “mental disorder” compared to the general population (14.86%, 1262/8494 vs 8.50%, 2103/24,738; *P*<.001), and for “supplementary factors” (11.80%, 1002/8494 vs 2.50%, 618/24,738; *P*<.001), the latter of which was largely driven by requests for contraception and contraception advice.

Table 3 provides an overview of visit outcomes for virtual visit users segmented by those seeing a known provider (seen before) or new provider (not seen before) compared to the matched control group with “traditional” visits. The likelihood of receiving a prescription was higher for virtual visit users than in-person visits (49.40%, 3599/7286 vs 45.69%, 10,043/21,981; *P*<.001). Referral to diagnostic imaging was lower for virtual visit users than in-person visits (1.88%, 137/7286 vs 6.59%, 1448/21,981; *P*<.001), but more likely if seeing a known provider (*P*<.001). Referral for laboratory testing was about the same for a virtual visit with a known provider (15.36%, 520/3385) and traditional visits (14.96%, 3289/21,981), but much lower (5.95%, 232/3901) for virtual visits with new providers (*P*<.001).

**Figure 3:**
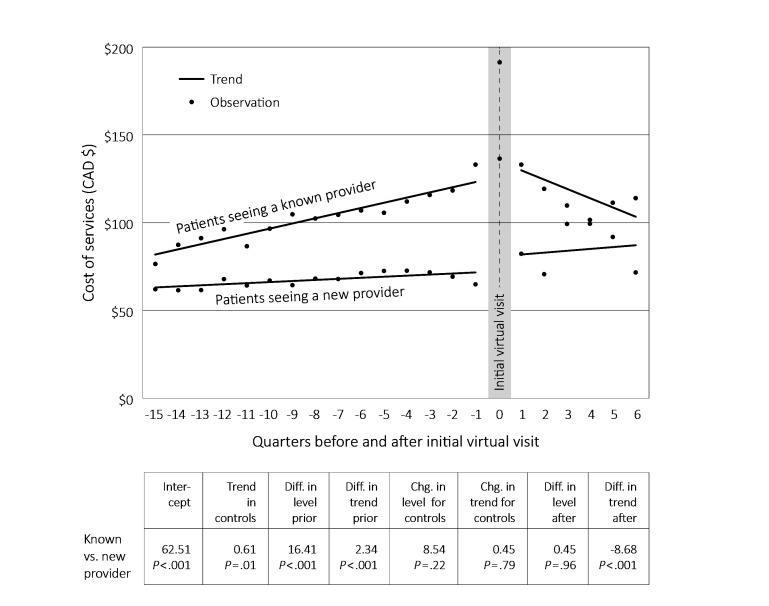
Time series analyses comparing virtual visit patients seeing a known provider to patients seeing a new provider, 2011-2014.

Figure 3 provides the interrupted times series results (regression parameter estimates) comparing patients seeing a known provider to patients seeing a new provider. Before the first virtual visit, the group who saw a known provider had both a higher cost (Can $16.41, *P*<.001) and a larger increasing trend in costs (Can $2.34, *P*<.001) compared to those who saw a new provider. After the intervention, the known provider group showed a decreasing trend (Can –$8.68, *P*<.001) compared to the new provider group, with the values approaching each other by the end of the follow-up period. Follow-up ended after six quarters because the numbers declined to the point that outcomes were not stable after this.

**Figure 4:**
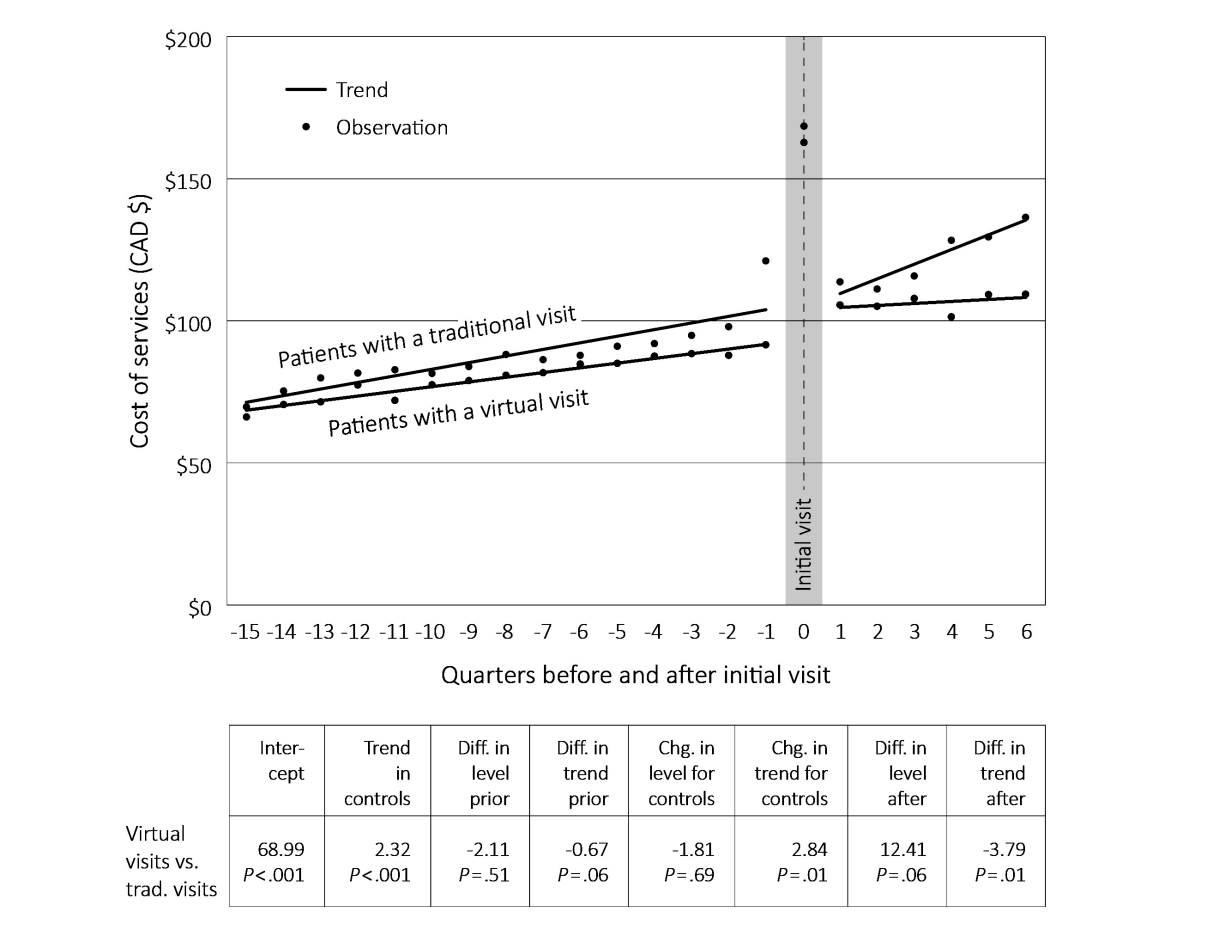
Time series analyses comparing patients with a virtual visit to matched controls with a traditional visit, 2011-2014.

Figure 4 provides the interrupted time series results (regression parameter estimates) of the total virtual visit group compared to a matched comparison group of people who did not have a primary care virtual visit during the study period, but who do had at least one traditional general practitioner (GP) visit. In this case, both groups showed an increasing trend in primary care spending before the GP visit. After the intervention, the patients with a virtual visit had a lower trend than their matched controls (Can –$3.79, *P*=.01). The result was an apparent lower expenditure among the virtual visit group at the end of the follow-up period, but again this period was limited. The trends were the same when the outcome was primary care visits rather than costs (data not shown).

##### Patient Survey

A total of 399 of 3025 (13.19%) BC residents who had a virtual visit in the past year completed the online patient survey from April 17 to May 1, 2015. The survey took a mean 18 minutes to complete. The majority of respondents, were female (71.4%, 285/399), between 35 and 54 years (45.1%, 180/399), and married (67.4%, 269/399). Population comparisons were provided along with sample demographics, health service utilization, and detailed results are available in the full report [29].

A number of aspects of the patient-physician engagement on the virtual visit were viewed positively, with 93.2% (372/399) reporting that their most recent visit was of high quality, 95.0% (379/399) reporting confidence in the security and privacy of their personal information when using a virtual visit, and 79.0% (315/399) saying their most recent virtual visit was as thorough as an in-person visit.

In terms of visit outcome, 91.2% (364/399) of respondents reported that the virtual visit was “very” or “somewhat” helpful to resolve health issue for which they needed the appointment. Only 1.5% (6/399) of patients reported that they were advised to call 9-1-1 or visit an emergency department immediately. Nearly half (48.4%, 193/399) of patients indicated they would have gone to a walk-in clinic if the virtual visit had not been available, 20.3% (81/399) would have had an in-person visit with their doctor or regular place of care, and 10.8% (43/399) would have gone to the emergency department. A total of 12.5% (50/399) reported that they would not have sought care at that time.

**Table 2:** Diagnoses for virtual visits, 2011-2014.

Diagnosis	Virtual visit users, n (%)	Traditional visit users (matched control), n (%)	*P*
Symptoms, signs, and ill-defined conditions	1299 (15.29)	4914 (19.86)	<.001
Mental disorders	1262 (14.86)	2103 (8.50)	<.001
Supplementary factors influencing health status and contact with health services	1002 (11.80)	618 (2.50)	<.001
Diseases of the respiratory system	852 (10.03)	3337 (13.49)	<.001
Diseases of the musculoskeletal system and connective tissue	540 (6.36)	1932 (7.81)	<.001
Diseases of nervous system and sense organs	525 (6.18)	1600 (6.47)	<.001
Endocrine, nutritional and metabolic diseases and immunity disorders	507 (5.97)	1387 (5.61)	<.001
Infections and parasitic diseases	467 (5.50)	1317 (5.32)	<.001
Diseases of the circulatory system	381 (4.49)	1418 (5.73)	<.001
Diseases of the genitourinary system	376 (4.43)	1662 (6.72)	<.001

**Table 3:** Number of visits resulting in a referral or other follow-up care for virtual and matched control visits, 2013/2014.

Referral/follow-up	Virtual visits (n=7286)				Matched control visits (n=21,981)	
	n (%)	Known provider, %	New provider, %	*P*	n (%)	*P*
General practice	113 (1.55)	2.22	0.97	<.001	314 (1.43)	.45
Medical specialists	125 (1.72)	1.60	1.82	.46	356 (1.62)	.58
Surgical specialists	78 (1.07)	1.18	0.97	.39	480 (2.18)	<.001
Imaging	137 (1.88)	2.90	1.00	<.001	1448 (6.59)	<.001
Laboratory test	752 (10.32)	15.36	5.95	<.001	3289 (14.96)	<.001
Prescription	3599 (49.40)	49.28	49.50	.85	10,043 (45.69)	<.001

## Discussion

Patients from all demographic and medical backgrounds are using virtual visits. Similar to other studies, younger patients are more likely to have a virtual visit compared to older individuals [3,4], perhaps emphasizing a digital divide between younger patients more comfortable with technology than older patients [30] or patients most likely to not have a regular care provider [31]. Patients with one or more major ADGs are more highly represented, which makes sense if these services are being used for monitoring existing conditions and providing services such as prescription refills that help patients avoid taking time and money to attend an in-person visit.

A small number of physicians are providing virtual visits. Male and female physicians are equally likely to be providing virtual visits, but there is a clear age gradient, with younger physicians far more likely to be using this new technology for patient care. In addition, we see that physicians who are identified as operating low-responsibility practices are significantly more likely to be providing virtual visits than those running high-responsibility practices. This suggests that at least some proportion (and likely a high proportion) of virtual visits may be essentially a virtual walk-in clinic [27].

Virtual visits were being provided in communities in British Columbia regardless of whether they were in urban and rural settings, indicating that virtual visits may not necessarily be filling all geographic gaps in primary care delivery [32]. Our analyses show that approximately one-third of people using virtual visits are seeing physicians with whom they previously interacted in a traditional office visit setting, whereas the remainder are seeing new providers. There were no sex differences in these percentages, but older people and those with more health problems were more likely to see a known provider. These appear to be positive trends from a patient care standpoint, consistent with monitoring of chronic conditions [33] and provision of patient-centered care [34]. Not seeing a known provider may reflect a desire for convenience over continuity [35], but may also indicate (at least in some cases) a specific preference for a new provider, such as for questions or care (eg, contraception) that patients wish to keep from their regular providers. Although there is no socioeconomic gradient in the use of virtual visits overall, individuals from lower income neighborhoods are less likely to see a known provider in their virtual visit.

The time series analyses comparing virtual versus traditional visits suggests that virtual visits may be beneficial in moderating total primary care costs over the longer term. At the same time, seeing a known versus new provider is better from an overall cost/use perspective. Putting these together, the conclusion is that virtual visits may have a beneficial effect—they are well-liked by patients (as seen in survey results) and they appear to control costs—but that benefit is most associated with seeing a known provider. Some caution is needed with these interpretations given the small N and limited follow-up period, but they are at least suggestive. The implication is that it matters (potentially quite a lot) *how* virtual visits are embedded in health care delivery systems.

This is consistent with previous research showing that virtual visits can complement existing patterns of care and, in fact, reduce overall primary and urgent care visits for patients [18,36]. Similarly, an evaluation by the US National Institute of Justice found that virtual care more generally (including synchronous and asynchronous care) has specific value for defined patient populations, reducing external visits to specialists and costly off-site transfers for care of prisoners [37] and providing a cost-effective solution for patients with limited access to mental health services [38].

### Limitations

Patient-initiated virtual visits in primary care are a relatively new model of access to primary care. Their use is growing rapidly, including in British Columbia, but is still a very small portion of total primary care. Although interrupted time series is a strong quasi-experimental analytic approach for evaluating population-level health interventions [39,40], it does not deal with selection bias and that may influence what we see in these analyses. Patients are not randomized to receive virtual visits, they choose to pursue them, and those who do and do not choose to use these visits may be different in unmeasured ways that are also related to primary care costs. Our comparison of users who see known versus new providers is further limited by the fact that these two groups are different in demographic and health status characteristics. Not all physicians choose to provide virtual visits, so selection at the physician level has some influence on which patients receive health care in this new format. These analyses consider only the costs of primary care. Future analyses should consider broader health care system effects of virtual visits and also the timing of virtual visits in longitudinal episodes of care.

### Conclusions

British Columbia is unique in Canada in offering publicly funded virtual visits in primary care. The province-wide implementation—both available for interactions among patients and physicians who know each other and for walk-in-type visits—makes this research valuable to other jurisdictions in Canada and beyond. The number of virtual visits is continuing to increase and it appears that there is no simple conclusion about their effect on existing patterns of health care services use. In some cases, the care is complementary, providing a new way for patients and providers with existing relationships to interact. In other cases, the care may be easing access, perhaps providing patients with needed care at a convenient point in time, but not necessarily displacing subsequent service use. A patient-centered system is one that is organized to respond to patient need without these unintended side effects. Virtual visits may be one means by which the system can be reoriented to be more patient-centered.

In the context of primary care transformation, there are discussions of new models of care and the role of technology in care, with the understanding that technology may be one way to improve care delivery [35]. Our analyses suggest that it is important to consider how such technologies are integrated into the system, whether as an adjunct to existing relationships or simply another way to see a provider on-demand. As technology continues to develop, it is important to ensure health care systems harness it to increase opportunities for patient-centered care.

## Abbreviations

EMR: electronic medical record
GP: general practitioner
HSDA: health service delivery area
ACG: adjusted clinical group
ADG: aggregated diagnosis group
SES: socioeconomic status
